# Primary Staphylococcal Lung Abscess: A Rare Situation in Infants

**DOI:** 10.7759/cureus.74303

**Published:** 2024-11-23

**Authors:** Amara Ayoub, Hanane Salhi, Zaari Najlae, Abdelouhab Ammor, Houssain Benhaddou

**Affiliations:** 1 Pediatric Surgery, Mohammed VI University Hospital, Oujda, MAR; 2 Pediatric Surgery, Centre Hospitalier Universitaire (CHU) Mohammed VI, Oujda, MAR

**Keywords:** abscess surgery, • lung abscess, mch pediatric surgery, pediatric thoracic surgery, primary lung abscess, staphylococcal lung abscess

## Abstract

Primary pulmonary abscess is a rare but serious localized bacterial infection of the lung parenchyma, occurring without prior lung conditions like bronchiectasis or necrotizing pneumonia. We report the case of an 11-month-old child with a 22-day history of productive cough and fever, unresponsive to initial antibiotics. Clinical examination showed a stable, eupneic child with mild fever and reduced oxygen saturation. Imaging revealed a large mass in the right upper lung lobe, necessitating surgical intervention. A thoracotomy confirmed a pulmonary abscess, and cytobacteriological analysis identified a resistant Staphylococcus spp. Postoperative recovery was successful following tailored antibiotic therapy. This case highlights the importance of comprehensive evaluation and timely surgical management in treating primary pulmonary abscesses in children.

## Introduction

A primary pulmonary abscess is defined as a localized and suppurative bacterial infection of the lung parenchyma. It is often caused by bacterial infections, with common pathogens including Staphylococcus aureus, Streptococcus pneumoniae, Klebsiella pneumoniae, and various anaerobes such as Bacteroides and Fusobacterium species, which occur independently of any antecedent pulmonary pathologies such as bronchiectasis or necrotizing pneumonia. Although infrequent, this condition is of considerable clinical importance due to its potential severity, requiring medical and surgical intervention. The etiology of primary pulmonary abscesses in children is frequently linked to the progression of untreated infection and the formation of a suppurative cavity within the lung parenchyma. The incidence of primary pulmonary abscess in infants and children is notably low, less than 1% of childhood pulmonary infections, thereby necessitating a comprehensive evaluation to exclude underlying etiologies before confirming a diagnosis of a primary abscess.

## Case presentation

An 11-month-old child with an unremarkable medical history was referred to the pediatric emergency department of our hospital. His medical history included a 22-day period of productive cough accompanied by fever, which led to his initial medical consultation and subsequent hospitalization for 12 days, during which he received antibiotic therapy amoxicillin-clavulanate. However, due to a lack of clinical improvement, further diagnostic assessments were deemed necessary.

Clinical examination on admission found a hemodynamically conscious, eupneic and stable child. His body temperature was recorded at 38.2°C, and his peripheral oxygen saturation (SpO2) was measured at 94%.

The pleuro-pulmonary examination indicated normal thoracic morphology without any signs of respiratory distress; however, diminished vesicular breath sounds were noted. Abdominal examination revealed a soft abdomen with no palpable masses.

**Figure 1:**
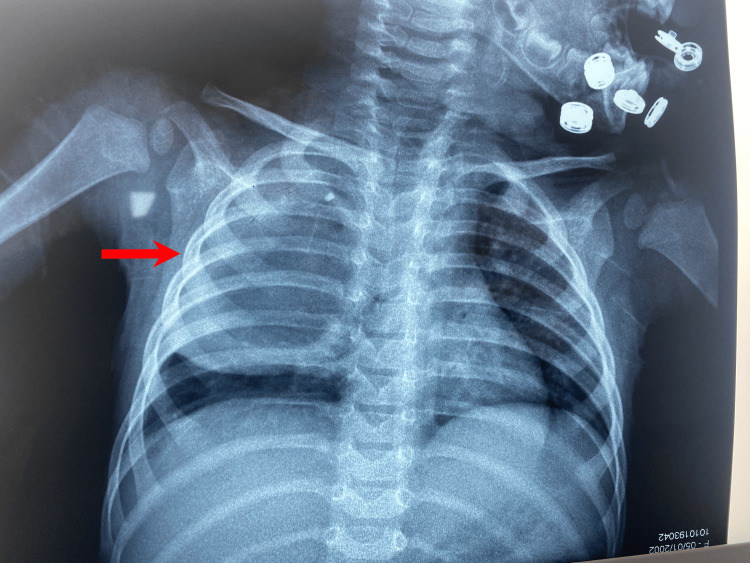
Frontal chest X-ray. The frontal chest X-ray reveals a well-defined opacity of water density, extending to the upper two-thirds of the right lung, without evidence of mediastinal displacement.

A chest X-ray was conducted which demonstrated a localized opacity in the right lung extending to the upper two-thirds of the lung without mediastinal displacement (Figure 1).

Subsequent thoracic angio-CT imaging revealed a substantial mass occupying the entirety of the right upper lobe. This mass contained both air and fluid compartments, with septa that enhanced following contrast administration. The septa's maximum thickness was measured at 2 mm. The mass exerted compressive effects on the adjacent lung parenchyma, though no wall calcification or bronchial fistula was discernible. The mass's dimensions were 72 x 67 x 46 mm (height x width x length). The anatomical relationships of the mass were as follows:

- Medially: The mass displaced the trachea, right main bronchus, upper lobe bronchus, superior vena cava (SVC), and azygos vein, the latter of which exhibited a reduced caliber but remained patent. Additionally, the superior lobar pulmonary artery, as well as the middle and inferior lobar pulmonary arteries and the right superior pulmonary vein, were displaced.

- Superiorly: The mass was in proximity to the right subclavian artery and the brachiocephalic trunk, delineated by a separation line.

- Laterally and anteriorly: The mass was in contact with the thoracic wall without evidence of bone lysis.

A moderate right-sided pleural effusion was observed, causing passive collapse of the lung and enhancement of the pleural layers. Mediastinal and axillary lymph nodes were also noted, measuring up to 9 x 8 mm.

**Figure 2:**
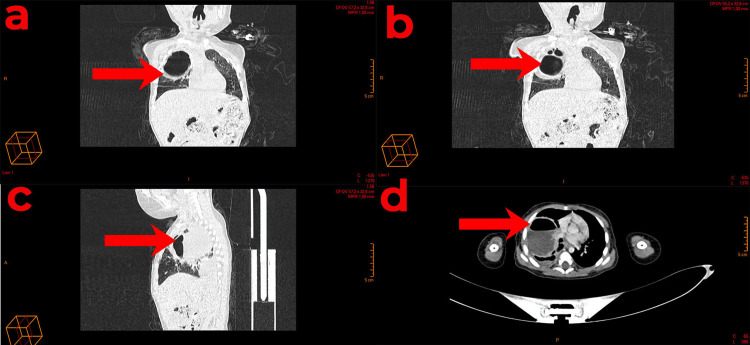
A computed tomography (CT) scan displaying a lung abscess (A, B) Coronal views, (C) a sagittal view, (D) a transversal view.

In conclusion, imaging revealed a substantial cystic mass on the right side of the lung, containing both air and fluid (Figure 2).

Upon admission, the patient's laboratory results indicated a C-reactive protein (CRP) level of 131 mg/L, a white blood cell count (WBC) of 15,990/μL (with neutrophils at 12,430/μL), a hemoglobin level of 6.4 g/dL, a prothrombin time (PT) of 97%, and a partial thromboplastin time (PTT) of 25 seconds.

Given the clinical presentation, the lack of response to intravenous antibiotic therapy, and the imaging findings, a decision was made to proceed with surgical intervention. The patient was subsequently transferred to the operating room.

**Figure 3:**
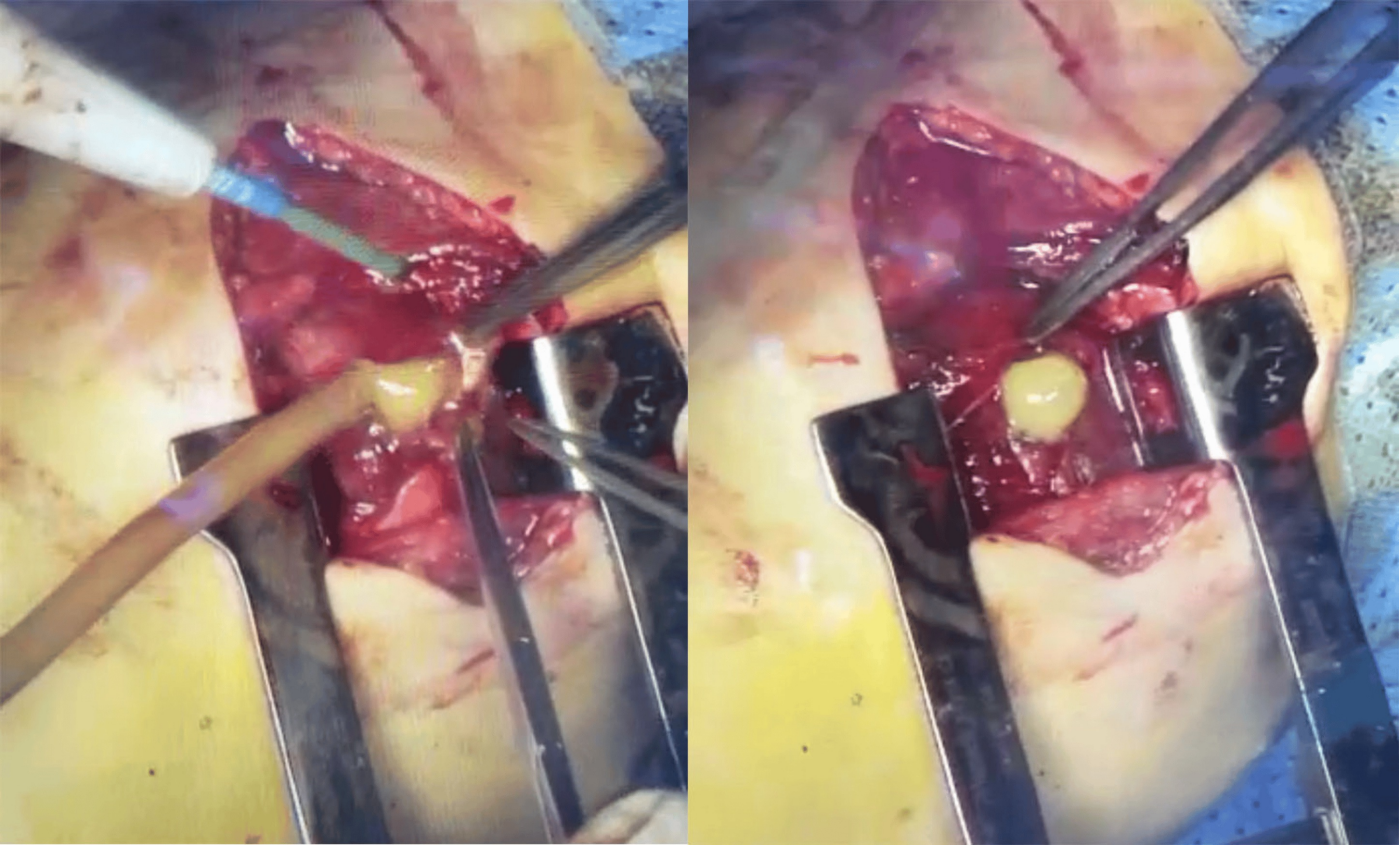
Intra-operative view. Puncture of the pulmonary abscess, yielding purulent fluid.

Under general anesthesia, with the patient in a lateral decubitus position, a posterolateral thoracotomy at the 4th right intercostal space was performed. Upon entering the fourth intercostal space, a right upper lobe pulmonary abscess encapsulated within a distinct capsule was identified. Aspiration of the abscess produced purulent fluid. The capsule was resected, and no fistula was detected during the ventilation test (Figure 3).

In the cytobacteriological analysis of the pleural fluid, the specimen exhibited turbidity, a brown hue, and a viscous consistency. Following Gram staining, the absence of bacterial flora was noted initially. However, after an incubation period of five days, bacterial growth was observed in the culture. The identified microorganism was determined to be Staphylococcus spp. Notably, this strain demonstrated resistance to penicillin G, ampicillin, fusidic acid, mupirocin, rifampicin, and linezolid.

**Figure 4:**
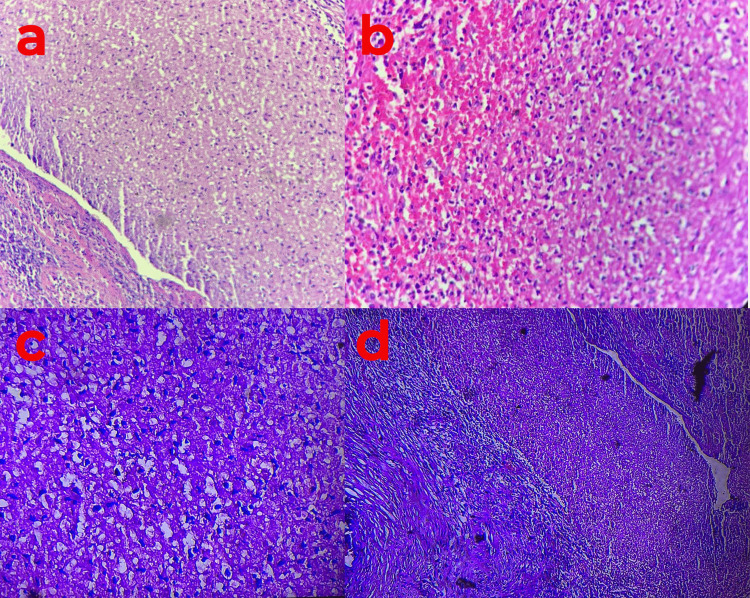
Pulmonary biopsy (a-d) The image depicts a microscopic view of extensively necrotic pulmonary parenchyma, exhibiting an inflammatory infiltrate predominantly comprised of neutrophilic polymorphonuclear leukocytes. The staining method utilized is Hematoxylin and Eosin, with a magnification of x400.

Upon anatomopathological examination of the abscess capsule, characteristic findings included suppurative inflammatory changes and necrotic alterations within the pulmonary parenchyma. Remarkably, the absence of epithelioid and giant cell granulomas was noted, as well as the absence of any malignant features (Figure 4).

**Figure 5:**
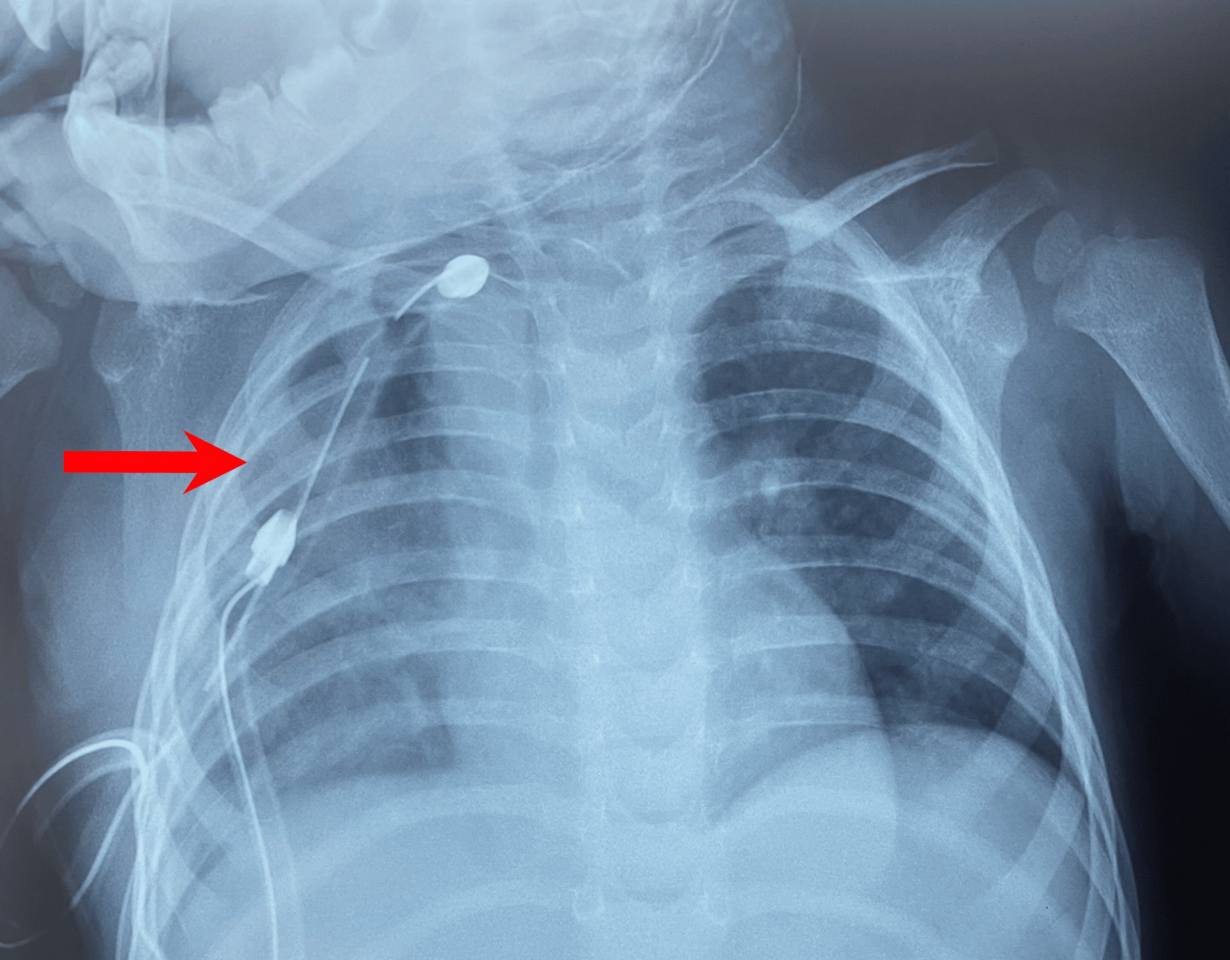
Frontal chest X-ray of the 3rd day post-operative.

**Figure 6:**
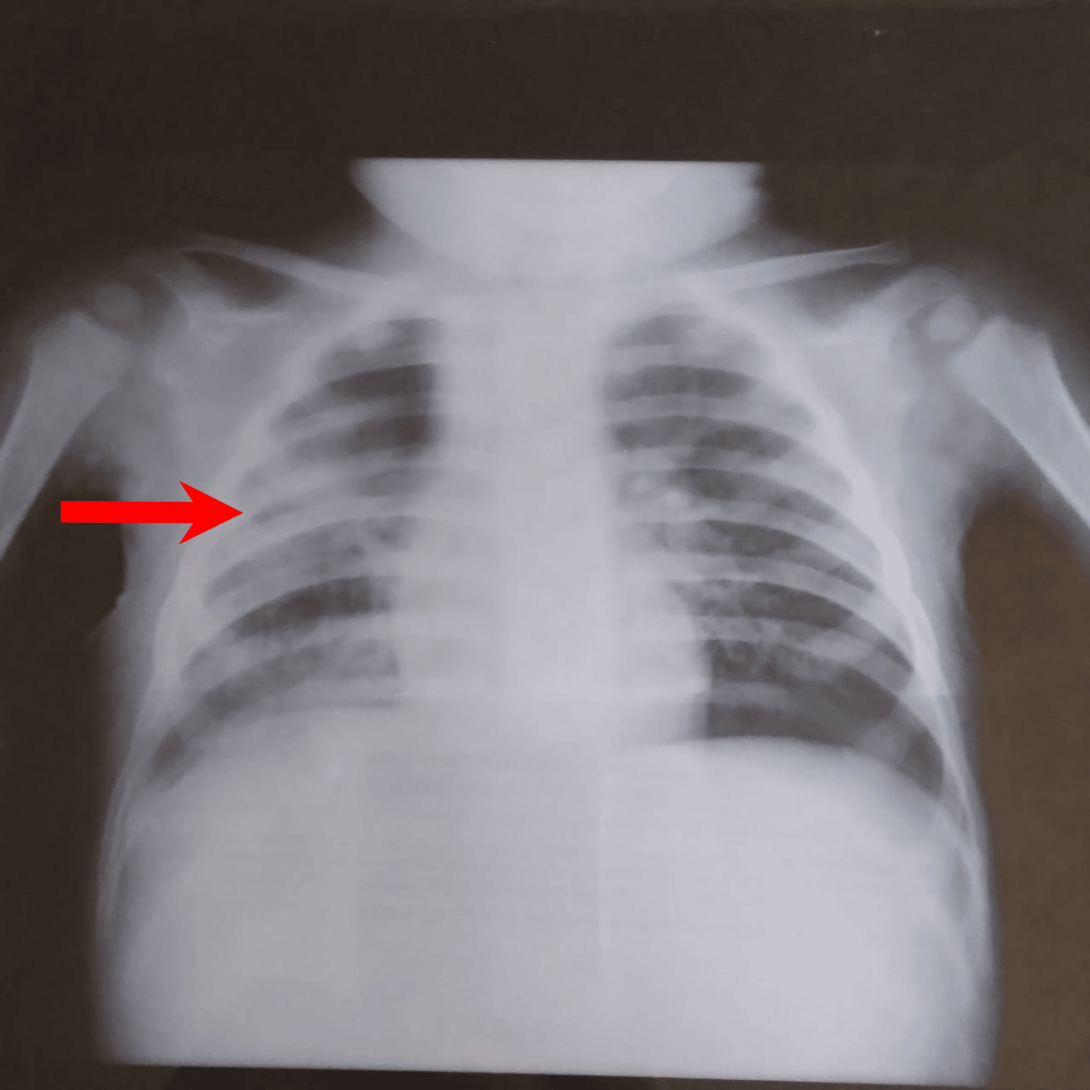
Frontal chest X-ray of the 2nd week post-operative.

**Figure 7:**
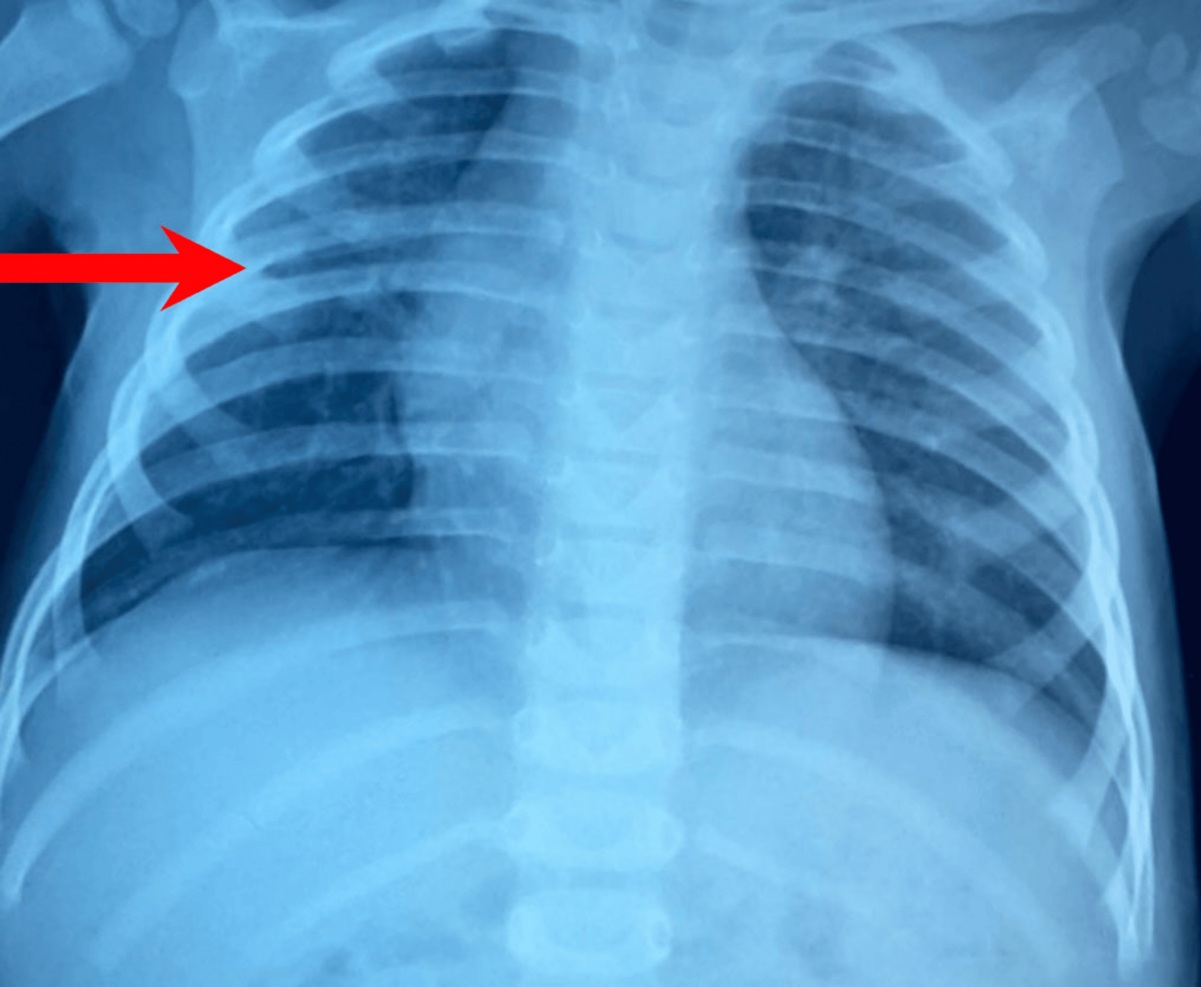
Frontal chest X-ray of the 8th month post-operative.

Regarding the clinical progression, the child exhibited stability in hemodynamics and respiratory status. Notably, the patient remained afebrile with a temperature of 37.2°C and maintained oxygen saturation levels at 98%. Drain removal occurred five days postoperatively, followed by the absence of fever within 24 hours. Initially, the patient was administered amoxicillin + clavulanic acid (Figure 5) with subsequent adjustments made to the treatment regimen following culture results by the amoxicillin-clavulanate for three weeks. The follow-up included blood tests for CRP levels and a chest X-ray (Figures 6, 7).

## Discussion

The pulmonary primary abscess represents a focal accumulation of pus or necrotic material within lung tissue, resulting in cavity formation. When accompanied by the development of a bronchopulmonary fistula, the cavity may exhibit an air-fluid level. Lung abscesses are categorized into primary and secondary types based on the presence or absence of pre-existing conditions [1]. A primary lung abscess manifests in an otherwise healthy child with normal lung function.

The absence of pleural syndrome on clinical examination and the lack of pleural thickening on chest radiography effectively eliminate the possibility of encapsulated pleurisy. While the patient's age might suggest a congenital bronchogenic cyst, the absence of complications or infection in this instance excludes such a diagnosis, a conclusion reinforced by imaging studies.

In endemic regions such as Morocco, consideration of a hydatid cyst is warranted. However, given the young age of patients, the presence of air-fluid levels and the absence of liver involvement on imaging, an infectious etiology is more probable.

Pulmonary tuberculosis is highly improbable at this age, particularly in the absence of prior exposure and given proper vaccination. If primary infection were to occur, it would typically present differently, with mediastinal lymphadenopathy and a tuberculous cavity lacking air-fluid levels [1-2].

Considering the patient's age, the possibility of an infected pulmonary malformation, such as cystic adenomatoid malformation, merits consideration. The presence of a pulmonary abscess does not necessarily rule out this diagnosis, even in the absence of a respiratory history [3].

Ultimately, considering the recent onset, clinical and biological signs of infection, and chest radiography findings, necrotizing pneumonia or a primary pulmonary abscess emerge as the most plausible diagnoses. Imaging and laboratory results confirmed this determination, further supported by histopathological examination. The absence of identifiable causes during the etiological investigation solidifies the diagnosis as primary. Notably, no specific neurological deficits, bronchopulmonary history, foreign body inhalation, underlying local causes, immune disorders, or cutaneous, vascular, or joint entry points were identified.

Clinical symptomatology is characterized by fever, cough, dyspnea, and anorexia. Additional signs, including gastrointestinal disturbances, purulent expectoration, and chest pain, are frequently observed. Hemoptysis is infrequent, and 17.4% of children exhibit associated acute otitis media. The absence of pathological history, particularly pulmonary, lends credence to the primary nature of the abscess [1-4].

Computed tomography (CT) serves as a valuable diagnostic tool, especially in challenging cases where distinguishing between a pulmonary abscess and a bronchogenic cyst is necessary. This modality is particularly useful for investigating underlying local causes or precisely delineating anatomical relationships before surgical intervention [5-6].

The most commonly identified pathogens in pediatric lung abscesses are Streptococcus pneumoniae and Haemophilus influenzae. Gram-negative bacilli and anaerobes are more frequently observed in cases of secondary lung abscess [1-7]. In the present case, the child presented with a lung abscess caused by Staphylococcus, a rarity in pediatric cases.

Initial treatment for primary lung abscesses typically involves intravenous antibiotic therapy targeting Staphylococcus, S. pneumoniae, and Gram-negative bacteria [8-9]. Failure of intravenous antibiotic therapy may necessitate surgical intervention if the abscess exceeds 6 cm in size. In such cases, options include chest tube insertion, pigtail catheter placement, thoracoscopy, thoracotomy, or lobectomy [2]. In this instance, open thoracotomy was chosen due to the dimensions of the abscess, enabling direct visualization and complete resection of the affected lung tissue.

The prognosis for lung abscesses remains guarded, particularly in cases of secondary origin. Pulmonary sequelae, such as bronchiectasis or pulmonary fibrosis, continue to pose a threat to one in six children [2-10].

## Conclusions

Lung abscesses represent a rare etiology of prolonged fever in children. Prior to ascribing a primary nature to such abscesses, a thorough investigation into local or systemic predisposing factors is imperative. Additionally, the role of surgical intervention should be carefully considered in cases of medical treatment failure. Long-term, meticulous monitoring of respiratory function remains paramount.
